# Establishing a Clinically Relevant Radiation Therapy Method for Preclinical Medulloblastoma Research

**DOI:** 10.1016/j.adro.2026.102114

**Published:** 2026-06-10

**Authors:** Jessica Buck, Meegan Howlett, Hilary Hii, Jacqueline Whitehouse, Zahra Abbas, Brooke Carline, Mani Kuchibhotla, Jacob Byrne, Jessica R. Lawler, Rebecca A. D’Alonzo, Eric Hau, Suki Gill, Nicholas G. Gottardo, Martin A. Ebert, Raelene Endersby

**Affiliations:** a Brain Tumour Research Program, WA Comprehensive Kids Cancer Centre, The Kids Research Institute Australia, Perth, Australia; b Centre for Child Health Research, Medical School, University of Western Australia, Nedlands, Australia; c School of Physics, Mathematics and Computing, University of Western Australia, Perth, Australia; d National Centre for Asbestos Related Diseases, University of Western Australia; and Institute for Respiratory Health, Perth, Australia; e Department of Radiation Oncology, Westmead Hospital, Sydney, Australia; f Westmead Institute of Medical Research, Westmead CLinical School, University of Sydney, Sydney, Australia; g Haematology and Oncology Service, Blacktown Hospital, Sydney, Australia; h Radiation Oncology, Sir Charles Gairdner Hospital, Perth, Australia; i Department of Paediatric and Adolescent Oncology/Haematology, Perth Children’s Hospital, Perth, Australia

## Abstract

**Purpose:**

Craniospinal irradiation (CSI) is a cornerstone of pediatric brain cancer therapy, yet detailed, reproducible protocols for accurate CSI delivery in preclinical mouse models remain scarce, hindering translational research and the development of radiosensitizing strategies. We aimed to establish a clinically relevant, easily replicable radiation therapy protocol for medulloblastoma (MB) mouse models, providing a robust platform for preclinical evaluation of novel therapeutic combinations. We hypothesized that multifraction CSI would most closely mirror clinical practice and achieve clinically representative disease control.

**Methods and Materials:**

Four radiation therapy strategies were compared to optimize treatment of orthotopic MB xenografts in mice: highly focal irradiation, regional brain targeting, whole brain exposure, and CSI. Mice received fractionated X-ray treatment using an X-RAD SmART 225Cx system. Survival was assessed by Kaplan-Meier analysis, recurrence patterns by histology, and DNA damage response activation by immunohistochemistry.

**Results:**

We evaluated 7 commonly used MB models (D283, D425, SU-MB002, MED813FH, MED211FH, TK-MB913, and a murine allograft). Focal irradiation provided minimal disease control, whereas regional brain targeting extended survival but failed to prevent rapid regrowth. Whole brain irradiation delayed progression but allowed spinal metastases. In contrast, multifraction CSI significantly improved survival across all models, with recurrence localized to the implantation site. Immunohistochemistry confirmed activation of DNA damage response pathways and cell cycle arrest, with distinct patterns between *TP53*-wildtype and *TP53*-mutated models.

**Conclusions:**

Multifraction CSI most accurately replicates clinical treatment and relapse patterns, outperforming focal and whole brain approaches. By defining CSI as the optimal strategy, we provide a clinically aligned, reproducible protocol with optimized dosing for common MB models, that will accelerate translational research and drive the development of next-generation radiosensitizers for pediatric brain tumors.

## Introduction

Medulloblastoma (MB) is the most common malignant brain tumor in children.^1^ Advances in understanding MB’s molecular features have led to its classification into 4 subgroups: Wingless, Sonic Hedgehog (SHH), Group 3 (G3) and Group 4 (G4); and further subdivision into 13 subtypes.^2^^,^^3^ Despite this, molecularly-directed therapies are yet to demonstrate clinical benefit and the standard of care remains surgery followed by radiation and multiagent chemotherapy.^1^^,^^4^ Accordingly, survival rates have stagnated for 30 years.

MB is metastatic; thus, the introduction of craniospinal irradiation (CSI) beyond local radiation therapy in the 1960s increased cure rates and became a treatment mainstay.^1^ Current radiation protocols are based on the Clinical and Molecular Risk-Directed Therapy for Newly Diagnosed Medulloblastoma trial (SJMB12), NCT01878617, where patients are stratified into low-, standard-, or high-risk groups by molecular subgroup and clinical factors and radiation therapy is delivered as daily 1.8-Gy fractions to reach the required cumulative dose.^5^^,^^6^ Standard-risk patients receive 23.4-Gy CSI in 13 fractions, whereas high-risk patients receive 36-Gy CSI in 20 fractions. The tumor bed then receives a conformal boost to a cumulative dose of 54 Gy.^5^^,^^6^

CSI is critical for MB treatment success; however, its use in pediatric patients causes debilitating, life-long toxicities.^7^, ^8^, ^9^, ^10^ Attempts to reduce CSI dose in a trial for standard-risk MB patients resulted in higher relapse rates and trial termination.^11^ Moreover, radiation therapy is typically delayed in children under 3 years to minimize toxicity; however, they experience shorter progression-free survival compared to older children receiving frontline CSI.^1^ This has prompted research efforts into radiosensitizing agents to enhance radiation efficacy and reduce side effects. Although preclinical literature is mounting, many studies fail to mimic clinical practice. Stone et al^12^ recently noted inadequate reporting of radiation timing, drug dosing and tumor size at treatment start, limiting reproducibility and clinical translation.

Recently, tools such as the X-RAD SmART 225Cx (Precision X-Ray) and the Small Animal Radiation Research Platform (SARRP, Xstrahl) have enabled image guided, conformal preclinical radiation therapy in small animals.^13^^,^^14^ When applied to closely mimic clinical radiation therapy within preclinical constraints, these tools are transforming radiation oncology research. With this in mind, we developed preclinical radiation therapy methods for treating MB mouse models using the X-RAD SmART 225Cx system. We considered similar methods developed for the SARRP^15^^,^^16^; however, system constraints prevented direct translation. Here we evaluate a range of methods using seven murine MB models to define optimal techniques. The CSI protocol delineated herein is comprehensive, relatively high throughput and elicits a biological response that closely paralleling clinical outcomes. This methodology can be readily integrated into preclinical workflows to advance future studies in radiation oncology.

## Methods and Materials

### MB cell lines and patient-derived orthotopic xenograft models

D425 and D283 human G3 MB cells were a gift from (Darell Bigner, Duke University, USA).^17^^,^^18^ SU-MB002 human G3 MB cells were a gift from (Yoon Jae Cho, Oregon Health and Science University, USA).^19^ Myc/p53^DD^ murine G3 MB cells were a gift from (Robert Wechsler-Reya, Columbia University, USA).^20^ Patient-derived G4 MB orthotopic xenograft (PDOX) TK-MB913 were developed at The Kids Research Institute Australia. These 4 cell models were modified to express firefly luciferase (Appendix E1). Patient-derived xenograft models MED813FH and MED211FH, derived from SHH and G3 MB respectively, were sourced from the Brain Tumor Resource Laboratory (Fred Hutchinson Cancer Research Center).^21^ Short tandem repeat analyses confirmed the identity of all human cell lines. Further details are provided in the Appendix E1.

### Intracranial implantation

Animal experiments were approved by The Kids Research Institute Australia Animal Ethics Committee, and conducted in accordance with the Animal Research: Reporting of In Vivo Experiments (ARRIVE) guidelines^22^ and the Australian Code for The Care and Use of Animals for Scientific Purposes. MB cells (10^5^) from culture (D425, D283, SU-MB002), or donor mice (Myc/p53^DD^, TK-MB913, MED211FH, MED813FH) were dissociated, suspended in Matrigel (BD Biosciences), and implanted into the right cerebral cortex or right cerebellum in 6- to 12-week-old mice (Appendix E1). Mice were randomized into treatment groups to achieve equivalent mean bioluminescence (BLI, measured as photons per second per square centimeter per steradian (p/s)) across groups; nonluciferase models were allocated randomly. Mice underwent weekly BLI and/or blood sampling. Survival was assessed using Kaplan-Meier analyses, with events defined as euthanasia due to brain or spinal tumor-related morbidity; non–tumor-related deaths were censored.

### Mouse strains and husbandry

NOD/*Rag1*^−/−^, NOD/*Rag1*^−/−^/*Il2rg*^−/−^ (NRG) and BALB/c nude mice were used for D425, D283, SU-MB002, TK-MB913, MED211FH, and MED813FH xenograft experiments. C57BL/6J/*Rag*1^−/−^ mice were used for the Myc/p53^DD^ allograft experiments. NRG mice were used for the toxicity assessment of the alternative CSI protocol described in the Appendix E1. Mice were aged 6 to 12 weeks at the time of tumor cell implantation. All mice had access ad libitum to food and water under a 12-hour light/dark cycle. Mice were maintained at the Bioresources Centre of The Kids Research Institute Australia. Further husbandry details are provided in the Appendix E1.

### Implantation site rationale

Implantation site selection reflected technical and biological considerations specific to each model. The cerebral cortex was used for initial experiments in cell line models because it provides a technically reproducible orthotopic site that yields consistent engraftment rates and predictable growth kinetics, enabling systematic comparison of radiation therapy strategies. The cerebellum, which is anatomically more faithful to the natural origin of MB, was used in later cohorts including patient-derived orthotopic xenograft (PDOX) models to verify that radiation therapy responses observed in cortical implants were not artifacts of implantation site, and to test the CSI protocol against tumors growing in the anatomically correct location. A summary of implantation site, cell number, mouse strain and sex, and treatment start day for each model is provided in Table E1.

### Radiation therapy protocols

Radiation therapy was delivered using an X-RAD SmART 225Cx system with SmART-Plan Monte Carlo-based dose planning software (Precision X-Ray). Irradiations were performed with a 225 kVp beam (0.32-mm Cu filtration, 1.0-mm Cu half-value layer [HVL]) with a large source size at 13 mA tube current, delivering approximate dose-rates of between 2.8 Gy/minute and 3.5 Gy/minute at isocenter. Determination of device output for absorbed dose calculations is made according to the protocol of the American Association of Physicists in Medicine using a calibration traceable to a primary standards laboratory.^23^ The X-RAD device and SmART-Plan systems are maintained via a routine quality assurance program. The spatial and dosimetric accuracy of the system has been reported.^24^ Treatment plans aimed to: (i) accurately calculate dose across the whole anatomy; (ii) minimize exposure to normal tissues; and (iii) ensure reproducibility within system constraints. Mice were anesthetized with isoflurane, positioned feet-first prone on a carbon-fiber stage, and cone beam computed tomography (CBCT) images acquired for dose planning. All multifraction schedules followed a 5-days-on, 2-days-off regimen, with a dose of 1.8 Gy per day. Additional details are provided in Appendix E1.

#### Dose prescription and uncertainty

For all 4 irradiation strategies, dose was prescribed such that at least 90% of the target volume (tumor or organ) received the prescribed fraction dose of 1.8 Gy. The total fraction dose was delivered cumulatively across the 2 opposed beams (square beam, whole brain, CSI) or 2 opposed arcs (focal protocol) within a single treatment session, with each beam or arc contributing approximately half of the prescribed dose at the target. Dose uncertainty at the isocenter was set at 2% within Monte Carlo dose calculations. The spatial and dosimetric accuracy of the X-RAD SmART 225Cx system at our institution has been characterized previously, with positioning accuracy of ±0.5 mm and dosimetric accuracy within 3% to 5% across the treatment field.^24^, ^25^, ^26^ The device is maintained via a routine institutional quality assurance program.

#### Focal irradiation

**Figure 1:**
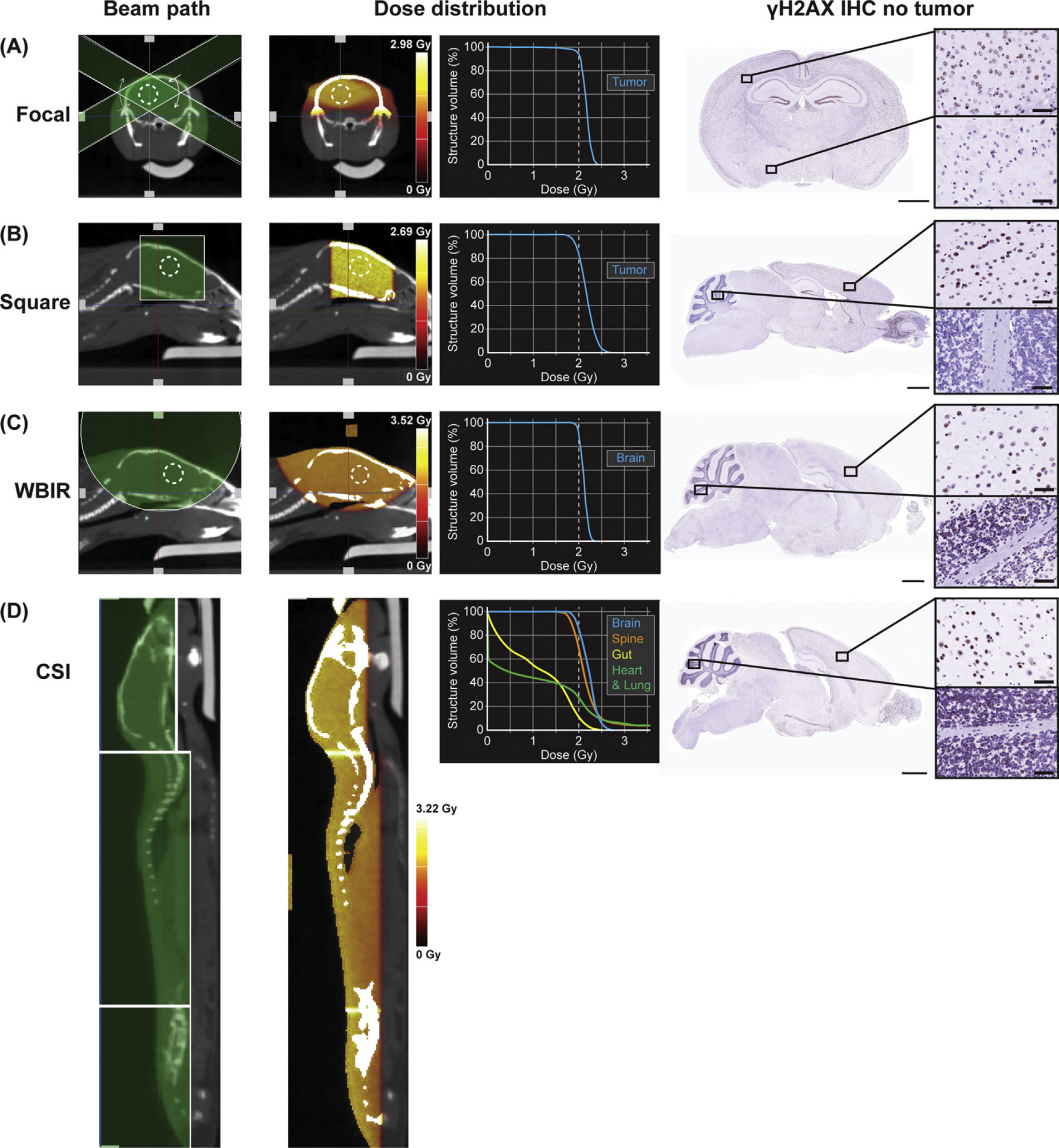
Treatment plans, dose distributions and validation of beam targeting for the four preclinical irradiation strategies evaluated in this study. Each row depicts 1 of the 4 irradiation strategies developed for the X-RAD SmART 225Cx system: (A) focal irradiation (5 mm circular collimation, 2 coplanar arcs); (B) square beam brain irradiation (10 mm square collimation, 2 lateral parallel-opposed beams); (C) whole brain irradiation (25 mm circular collimation, 2 lateral parallel-opposed beams); and (D) craniospinal irradiation (40 mm square collimation, 3 sets of lateral parallel-opposed beams down the length of the animal). Column 1 (beam path): CBCT cross-sections (coronal for focal; sagittal for square beam, whole brain and CSI) overlaid with treatment beam positions (*green shading*); white arrows in the focal panel indicate the direction of arc rotation. White areas demarcate bone; the area surrounding the implantation site (where applicable) is marked with a *dashed line*. Column 2 (dose distribution): dose–volume histograms (right) and corresponding dose heat maps overlaid on CBCT images (left) for each treatment plan, demonstrating delivery of ≥1.8 Gy per fraction to ≥90% of the target volume. Column 3 (γH2AX validation): IHC for γH2AX (*brown*, with hematoxylin counterstain in *blue*) on non–tumor-bearing brain performed 2 hours after a single 1.8-Gy fraction. Scale bars represent 2 mm in the low-magnification panels and 50 µm in the insets. Insets show representative areas within the treatment field (*top right* panel in A-B; *both panels* in C-D) and outside (*lower right* panels in A-B), demonstrating accurate beam targeting in each case (>90% γH2AX-positive nuclei within the beam path versus <5% outside). *Abbreviations:* CBCT = cone beam computed tomography; CSI = craniospinal irradiation; IHC = immunohistochemistry.

Two coplanar arcs with 5-mm circular collimation were delivered to a single isocenter located at the tumor implantation site, which was identified by skull landmarks visible in the CBCT images. The first (left lateral) beam rotated in an arc from 60° to 120°, and the second beam (right lateral) rotated in an arc from 150° to 210° (gantry angles commencing at 0° for a beam pointing vertically down and increasing with counter-clockwise rotation as seen in the tube cathode-anode direction) (Fig. 1A).

#### Square beam brain irradiation

Two lateral coplanar, parallel-opposed beams with 10-mm square collimation were delivered to a single isocenter centered on the cortex. The isocenter was positioned to minimize irradiation of the soft palate, olfactory bulbs, and brain stem (Fig. 1B).

#### Whole brain irradiation

Two lateral coplanar, parallel-opposed beams with 25-mm diameter circular collimation were delivered, with the brain positioned toward the edge of the collimated field, to avoid irradiation of the oral cavity (Fig. 1C).

#### Craniospinal irradiation

Anesthetized mice were gently secured to the stage using nonstick sports tape to flatten the spine and facilitate spinal irradiation. Three sets of lateral coplanar, parallel-opposed beams with 40 mm square collimation were delivered, with the animal occupying the lower portion of the field, such that abdominal organs are spared irradiation. The first target was placed above the head, the second 40 mm inferior above the middle of the abdomen, and the third a further 40 mm inferior above the base of the tail (Fig. 1D).

### Tissue collection and histology

To enable assessment of active DNA synthesis, BrdU (#B23151, Invitrogen, 2 mg/mouse) was administered intraperitoneally 1 hour before euthanasia. Animals were anesthetized and perfused with phosphate buffered saline (PBS) followed by 4% paraformaldehyde. After 16-hour fixation in 4% paraformaldehyde (Sigma) in phosphate buffered saline, brains were dehydrated and paraffin-embedded using standard protocols. Sections (5 μm) were stained with hematoxylin and eosin. For immunohistochemistry (IHC), citrate antigen retrieval preceded overnight incubation with primary antibodies (Cell Signaling Technologies) for γH2AX (#9718S, 1:500), cleaved caspase-3 (#9664S, 1:500), phospho-Histone H3 S10 (#9714S, 1:150), phospho-CHK1 S345 (#2348L, 1:200), phospho-CDC2 Y15 (#4539L, 1:200), and BrdU (#5292S, 1:200). Visualization used an Elite ABC kit with NovaRED substrate and Gill's hematoxylin counterstain (Vector Laboratories).

Quantification was performed using a Nuance Multispectral Imaging System and inForm software (Perkin Elmer) on at least 3 representative images per tumor. Spectral unmixing was first applied to separate the chromogen (NovaRED) signal from the hematoxylin counterstain. A supervised classifier was trained on the hematoxylin signal alone to segment tumor from adjacent normal brain tissue, and subsequently to segment individual nuclei within the tumor region; because this classifier relies only on hematoxylin (which is consistent across all samples), the same trained classifier was applied to all images across all markers and experiments. Each segmented nucleus was then classified as positive or negative for the marker of interest using intensity thresholding. As the level of background NovaRED staining varies between primary antibodies, thresholds were set visually for each marker against representative positive and negative cells, and the same threshold was then applied uniformly across all images for that marker within an experiment. Positive cells were expressed as a percentage of total nuclei within the tumor region and normalized to the mean of untreated controls within the same experiment.

### Statistics

Statistical analyses were performed using GraphPad Prism v9.1.0. Kaplan-Meier survival curves were compared using the Mantel-Cox log-rank test. IHC results were compared using 1-way analysis of variance with Dunnett’s multiple comparison test against untreated controls.

Sample size was guided by power analysis: cohorts of at least 4 mice per group were determined to provide 80% power to detect a 30% increase in survival between groups at α = 0.05 (further detail in Appendix E1). Variation in cohort size across experiments reflects: (i) initial pilot experiments using smaller cohorts (typically n = 5) to establish feasibility and approximate effect sizes for each new model and treatment combination; (ii) confirmatory experiments and primary radiation therapy comparisons using larger cohorts (n = 8-19) where statistical power was prioritized; (iii) constraints on tumor take rates, animal availability, and donor mouse yield, particularly for PDOX models; and (iv) adherence to the 3Rs principle and our institutional Animal Ethics Committee approval, which require the minimum number of animals consistent with scientifically meaningful results.

## Results

### Confining radiation to the site of implantation fails to control MB growth in vivo

To refine preclinical radiation therapy methods for MB models, we developed a reproducible, fractionated regimen mimicking clinical MB outcomes. It aimed to prolong survival, but not cure mice with intracranial G3, G4, or SHH MB, while limiting harm to healthy brain tissue and oral mucosa. Two conservative approaches were taken initially; focal (tumor-targeted), and square beam (wider margin), with both delivering 1.8 Gy per fraction to the implantation site. Accurate targeting was confirmed by immunohistochemistry whereby over 90% of nuclei within the beam path were γH2AX-positive, 2 hours after irradiation, compared to under 5% in off-target areas (Fig. 1A, B).

**Figure 2:**
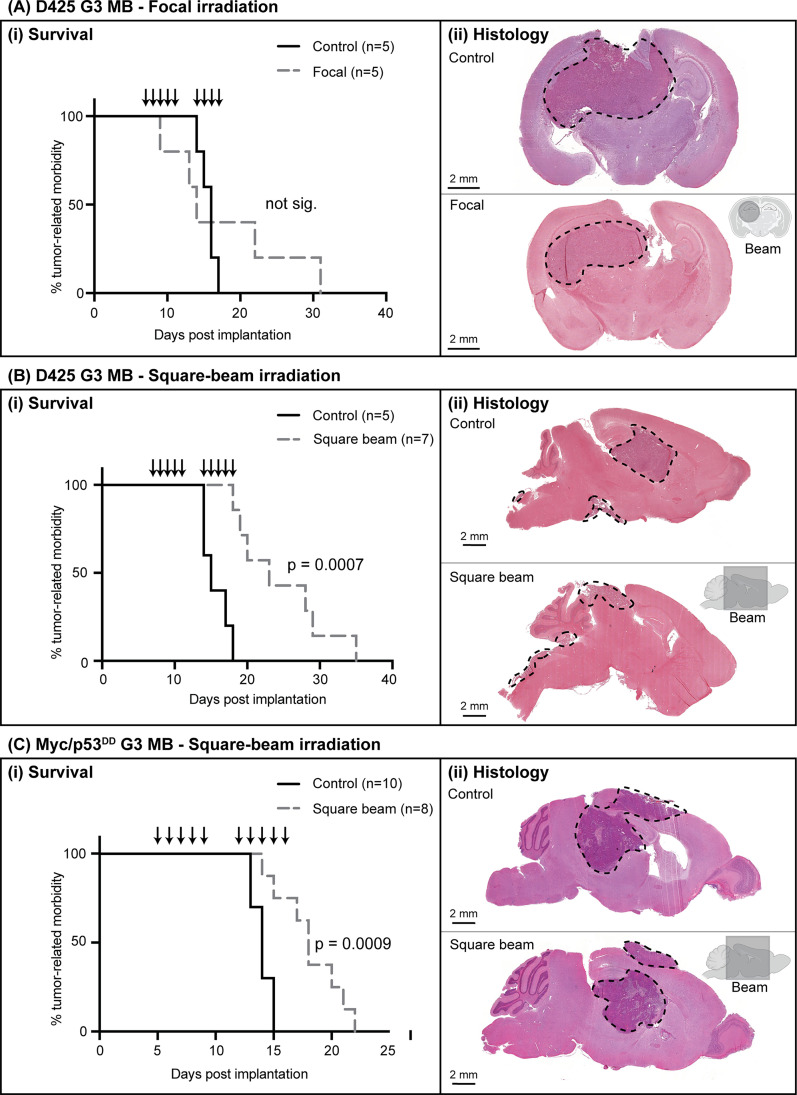
Focal and limited-field irradiation methods do not control tumor growth in orthotopic human xenograft or mouse allograft models of G3 MB. D425-bearing mice were treated with (A) focal irradiation or (B) square beam irradiation, and (C) Myc/p53^DD^-bearing mice were treated with square beam irradiation. Each panel shows (i) Kaplan-Meier survival curves for control or treated mice. Days of treatment are indicated by downward arrows and number of mice (*n*) are indicated. (ii) Representative H&E-stained sections of brains at the humane endpoint in untreated control or irradiated mice. Tumors are indicated (*dashed line*) and insets illustrate the treatment field (*dark grey*). *Abbreviations:* H&E = hematoxylin and eosin; MB = medulloblastoma.

Mice implanted with D425 G3 MB began treatment when tumor burden was low (7 days postimplantation), to mimic the postsurgical situation of patients. However, all mice receiving focal irradiation developed tumor-related morbidity while undergoing treatment, limiting the cumulative dose to 16.2 Gy (9 fractions). Treatment had no impact on overall survival (median survival 16 days control vs 14 days focal; n = 5 per group). Histologic assessment revealed tumors growing within and outside the irradiated area in all samples assessed (n = 4 per group) (Fig. 2A).

In attempts to better control tumor growth, the treatment field was expanded to a 10 × 10-mm area (Fig. 1B). D425 MB xenograft and Myc/p53^DD^ allograft mice were again treated with 1.8 Gy/day; however, treatment ceased after a cumulative dose of 18 Gy because of the onset of tumor-related morbidity. Treated mice exhibited statistically significant, but minimally improved survival (D425 median survival, 15 days control, n = 5 vs 23 days irradiated, n = 7; *P* = .007; Myc/p53^DD^ median survival, 14 days control, n = 10 vs 18 days irradiated, n = 8; *P* = .009). Histologic assessment of a subset of mice showed that at least half of the animals had tumor growth within the irradiated area. In both models, disease was also observed outside the treated area, such as olfactory bulb and meninges (Fig. 2B, C). These data reflect the highly metastatic nature of MB and indicate that these methods of preclinical irradiation are insufficient to control disease. Of note, no dose-limiting toxicities were observed using either method.

### Whole brain irradiation increases survival in mouse models of MB

**Figure 3:**
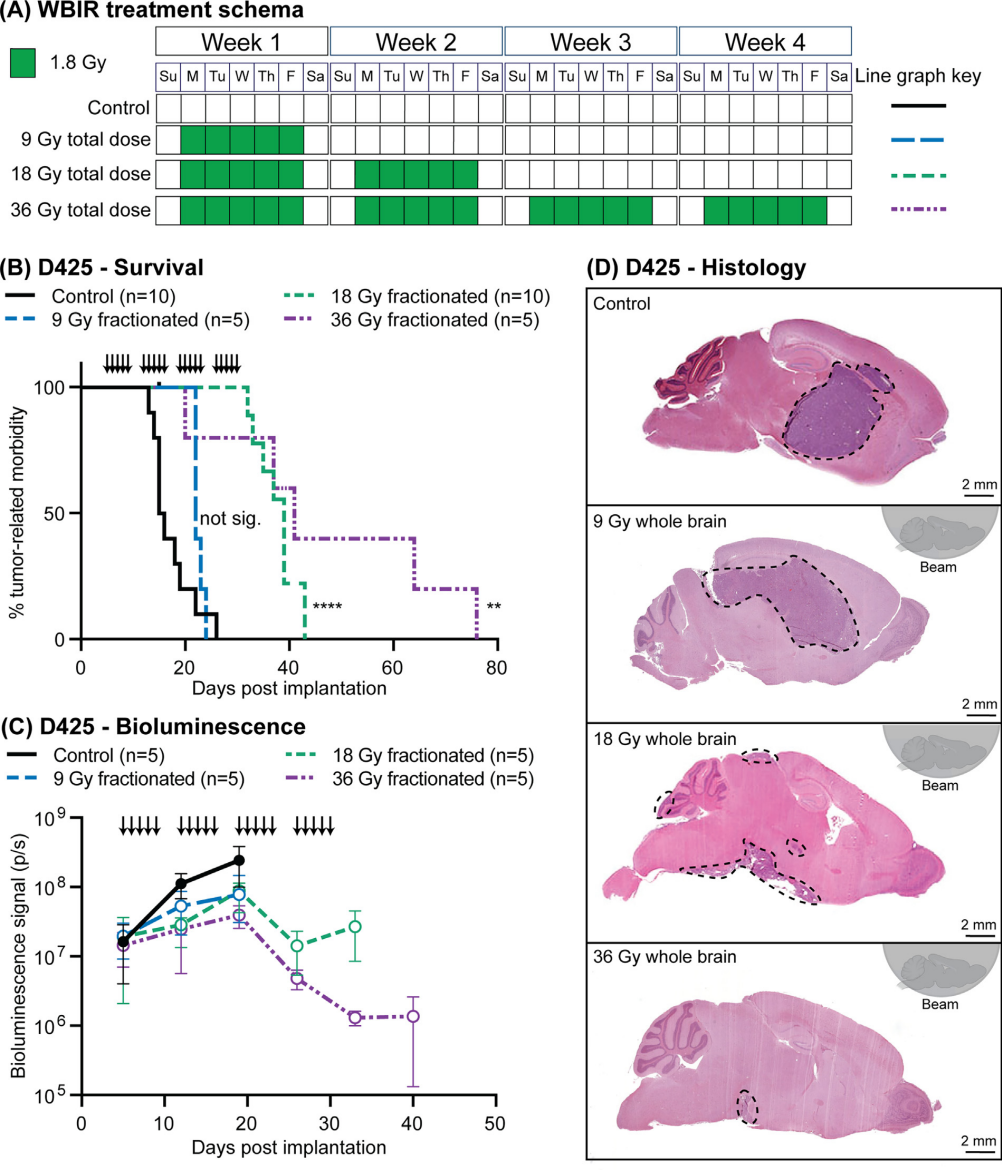
WBIR increases survival of mice with D425 MB. (A) Treatment schema. (B) Kaplan-Meier survival curves for control mice implanted with D425 cells or mice treated with 9, 18, or 36 Gy WBIR (arrows indicate treatment days, n indicates number of mice). (C) Bioluminescence measured from the heads of a subset of mice over time. (D) Representative H&E-stained sections of D425 tumors from control mice, and mice following 9-, 18-, or 36-Gy fractionated WBIR, at humane endpoint. Tumors are indicated (*dashed line*) and each inset illustrates the treatment field (*dark grey*). *Abbreviations:* H&E = hematoxylin and eosin; MB = medulloblastoma; WBIR = whole brain irradiation.

To better control MB growth in mice, we expanded the irradiation field to target the whole brain and tested increased cumulative doses. A 25-mm circular collimator was used where the beam was positioned to spare the soft palate and avoid oral mucositis.^27^ Dosimetry calculations and γH2Ax IHC confirmed accurate targeting (Fig. 1C). D425 MB xenograft mice received cumulative doses of 9, 18, or 36 Gy, delivered in 5, 10, or 20 fractions of 1.8 Gy (Fig. 3A). Nine gray whole brain irradiation (WBIR) was insufficient to extend survival compared to control mice (Fig. 3B; median survival 15.5 days control, n = 10 vs 22 days irradiated, n = 5). In contrast, cumulative doses of 18 Gy or 36 Gy significantly extended survival compared to controls (median survival 39 days, n = 10; *P* < .0001, or 41 days, n = 5; *P* = .002, respectively, vs control). Of note, irradiation of the whole brain resulted in significantly improved survival compared to when the same dose was delivered using the 10 × 10-mm square field (Fig. 2B vs Fig. 3B, median survival for WBIR 39 days, n = 10, vs 23 days using a square beam, n = 7; *P* = .0001). BLI revealed that 9-Gy WBIR did slow tumor growth, however decreased photon flux following 18- and 36-Gy irradiation suggests the higher doses induce tumor regression (Fig. 3C).

Despite irradiation, all mice eventually required euthanasia due to tumor progression. After 9 Gy irradiation, tumors were observed growing at the implantation site (Fig. 3D). In the other irradiated groups, only small tumors were found at the implantation site; however leptomeningeal spread was observed in 3 out of 8 mice after 18-Gy irradiation and in 3 out of 5 after 36-Gy irradiation. Tumors also frequently appeared at the cranial floor, which is possibly because of dose variation in this area from collimator-induced penumbra (Fig. 3D). Notably, despite 18- to 36-Gy WBIR successfully restricting intracranial tumor growth, all these mice developed spinal metastases, evident by hunched spines, hind limb paralysis, and confirmed by BLI (Figure E1).

To verify these findings, similar experiments were performed in mice implanted in the cerebellum with SU-MB002 G3 MB cells. In this model, 18-Gy WBIR significantly extended survival compared to controls (Figure E2, median survival 22 days in controls, n = 5 vs 51 days with WBIR, n = 5; *P* = .0011). Consistent with the other models, SU-MB002 MBs grew both at the implantation site and metastatically, with BLI indicating a rapid increase in tumor growth on treatment cessation. Leptomeningeal disease was detected in 4 out of 5 and hind limb paralysis was observed in 2 out of 5 mice, indicating that failure to treat the entire cranio-spinal axis is insufficient (Figure E2). No dose-limiting toxicities were observed during or following WBIR; mice in WBIR cohorts reached humane endpoints due to spinal metastasis-related morbidity.

### Craniospinal irradiation increases survival and mimics clinical treatment outcomes

Leptomeningeal disease and spinal metastases indicated the need to preclinically irradiate the entire craniospinal axis, as is performed clinically. Initially, we developed a CSI technique combining the WBIR protocol above, with irradiation of the spine using a 5-mm circular collimated beam delivered perpendicular to the dorsal axis from above the animal, similar to Morrissy et al^15^; however, severe adverse health effects were observed and this approach was not used further (Appendix E1, Figure E3).

Instead, we developed a method based on Smith et al^16^ Treatment was delivered using 2 40 × 40-mm square collimated beams from each side, in 3 fields down the length of the mouse. Exposure of normal tissues, especially abdominal organs was avoided by collimator positioning (Fig. 1D). γH2AX IHC confirmed accurate targeting of the entire brain. Using this approach, a cumulative dose of 18- to 20-Gy CSI was achieved with minimal adverse effects (Figure E4).

**Figure 4:**
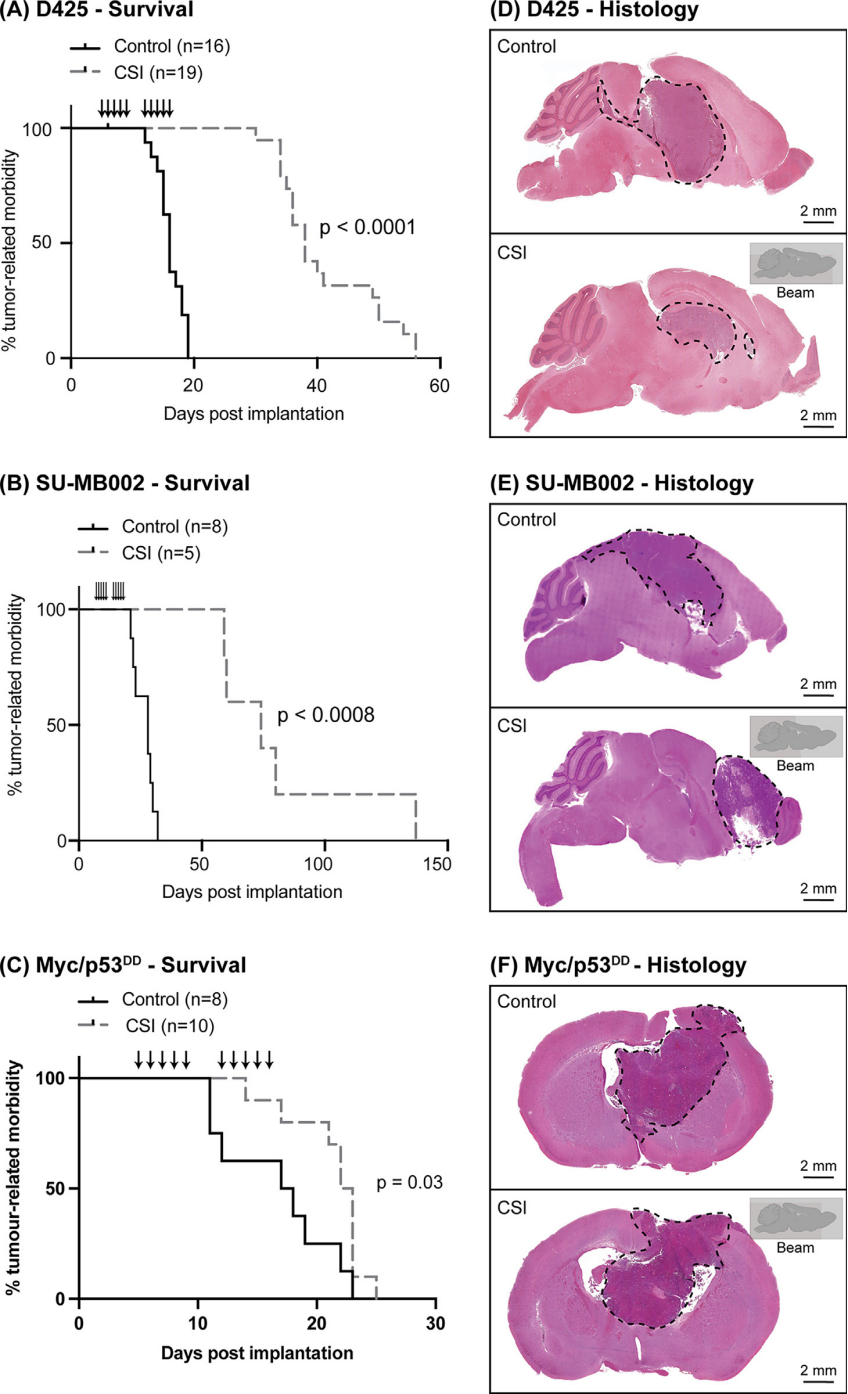
CSI increases survival of mice with G3 MB and effectively controls spinal metastasis. (A-C) Kaplan-Meier survival curves for mice implanted cortically with (A) D425; (B) SU-MB002; or (C) Myc/p53^DD^ cells treated as controls or with 18 Gy CSI (arrows indicate treatment days, *n* indicates number of mice). (D-F) Representative H&E-stained sections from the indicated models showing that on euthanasia, tumor growth (*dashed line*) was observed around the implantation site and/or within the treatment field (insets, *dark grey*). *Abbreviations:* CSI = craniospinal irradiation; H&E = hematoxylin and eosin; MB = medulloblastoma.

To evaluate this CSI protocol, mice were implanted with human or murine G3 MB cells. Significantly extended survival was observed in mice treated with 18-Gy CSI compared to controls across all models: D425 (Fig. 4A; median survival 16 days control, n=16 vs 38 days CSI, n = 19; *P* = .0001), SU-MB002 (Fig. 4B; median survival 28 days control, n = 8; vs 74 days irradiated, n = 5; *P* = .0008), and murine Myc/p53^DD^ MB tumors (Fig. 4C; median survival 17.5 days control, n = 8 vs 22.5 days irradiated, n = 10; *P* = .03). Notably, 18 Gy CSI did not cure any mice, mimicking clinical outcomes. For mice harboring D425 MB, the survival benefit of 18-Gy CSI was similar to 18-Gy WBIR (median survival 38 days in both groups, Figs 4A and 3B, respectively); whereas, for mice implanted with SU-MB002, CSI was superior (median survival 74 days with CSI vs 51 days with WBIR, Fig. 4B and Figure E2, respectively). Of note, across all 3 models, morbidity was caused by tumor growth around the site of implantation (Fig. 4D-F) with no evidence of spinal metastases, demonstrating effective control of metastatic disease.

This method of delivering fractionated CSI to mice was fast and efficient, at approximately 10 minutes per mouse to set up and treat, facilitating large preclinical experiments. Moreover, this method extends survival of murine G3 MB models without eradicating disease and thus is an ideal protocol for the investigation of radiosensitizing agents; as such, higher CSI doses were not assessed.

### Preclinical MB models exhibit a broad range of sensitivities to CSI

**Figure 5:**
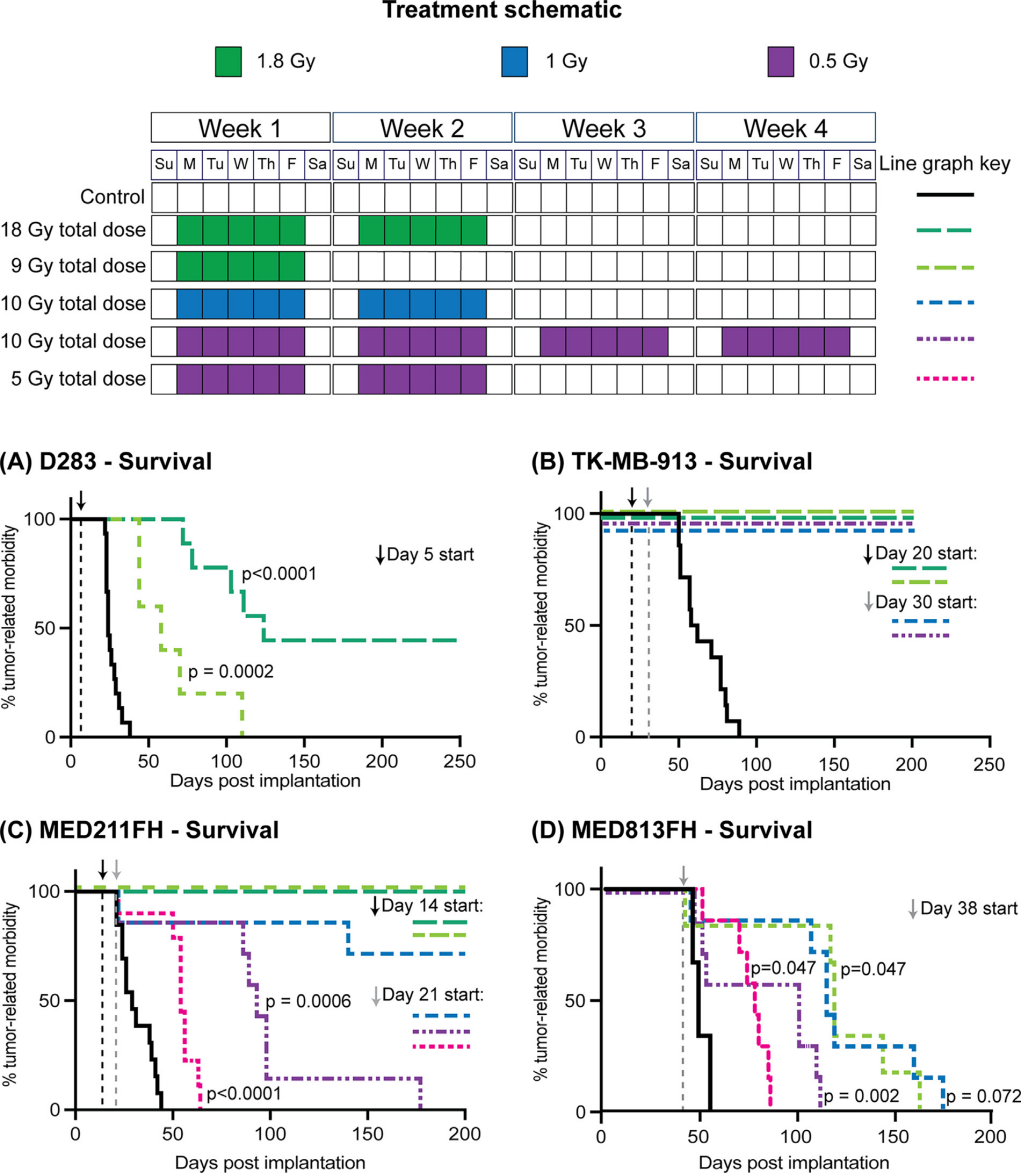
CSI dose optimization for PDOX models of MB. Treatment schema showing the different CSI doses and schedules evaluated is shown at top. (A-D) Kaplan-Meier survival curves for control mice and mice receiving varying doses of CSI are shown for (A) D283, (B) TK-MB-913, (C) MED211FH, and (D) MED813FH PDOXs. The treatment start day is indicated on each graph as follows: black arrows/dashed lines indicate treatment initiation in asymptomatic mice, grey arrows/dashed lines denote treatment initiation was delayed until mice exhibited early phenotypic changes associated with tumor growth. Groups were compared using log-rank tests relative to nontreated controls. *Abbreviations:* CSI = craniospinal irradiation; MB = medulloblastoma.

To determine how reproducible and/or suitable the effects of 18-Gy fractionated CSI are, the protocol was further evaluated using additional MB models. Unsurprisingly, marked differences in radiation sensitivity were observed. D283 human G3 MB cells were very sensitive to 18-Gy CSI, with 50% mice exhibiting long-term survival and no detectable disease for up to 250 days postimplantation (Fig. 5A; median survival 24 days control, n = 15 vs 124 days irradiated, n = 10; *P* < .0001). Instead, for D283 implants, 9-Gy CSI delivered in five 1.8-Gy fractions was more suitable, as this extended survival and also controlled metastatic disease given all mice required euthanasia for intracranial, not spinal, tumors (Fig. 5A; *P* = .0002; median survival 24 days control, n = 15 vs 58 days irradiated, n = 5).

Slower growing PDOX models are considered the gold standard tool for preclinical testing.^28^ We therefore tested a range of CSI protocols across numerous G3 and G4 MB PDOX models. Notably, 2 MB PDOX models were exquisitely radiosensitive. TK-MB913 PDOXs, representative of G4 MB, were completely eradicated with 9-Gy and 18-Gy CSI (delivered in 1.8-Gy fractions) (Fig. 5B). Although response may be determined by genetic factors, we queried whether tumor burden at treatment initiation may affect outcome. Therefore, we tested different cumulative doses, different doses per fraction and different treatment initiation days (Fig. 5, *top*) in TK-MB913 implanted mice. Measuring the effect of CSI in mice with higher tumor burden is challenging as these PDOX models do not persistently express luciferase even with lenti/retroviral transduction. Instead, treatment initiation was delayed until mice began exhibiting early phenotypic changes associated with tumor growth. Even with lower CSI doses and starting with larger tumors, all protocols were curative in this model (Fig. 5B). Similarly, for the MED211FH PDOX model representing G3 MB, mice were cured using 9-Gy CSI (delivered in 5 × 1.8-Gy fractions, Fig. 5C). Lower dose per fraction and later start times were also tested here; and while 10 Gy CSI delivered in 10 × 1-Gy fractions cured 71% of mice with MED-211FH, other schedules delivering 10-Gy or 5-Gy CSI were shown to extend survival without curing all mice (Fig. 5C; 10-Gy CSI, delivered in 20 × 0.5-Gy fractions, median survival 93 days, n = 7; *P* = .0006; 5-Gy CSI, delivered in 10 × 0.5-Gy fractions, median survival 54 days, n = 10, *P* < .0001; where median survival of controls was 29 days). As expected, 5-Gy CSI and schedules using a lower dose per fraction was less effective.

MB cases with *TP53*-mutated SHH disease harbor especially poor prognosis and require novel clinical approaches. To optimize preclinical radiation therapy for PDOX models representing this subtype, we evaluated different CSI protocols in the MED813FH PDOX model. All schedules significantly extended survival without curing any mice (Fig. 5D). Median survival of control animals (48 days), was extended to 77 days with 5-Gy CSI (10 × 0.5-Gy fractions; *P* < .01). More effective was 10-Gy CSI, tested in 3 different schedules of 20 × 0.5 Gy, 10 × 1 Gy, or 5 × 1.8 Gy, which extended median survival to 100 (*P* < .05), 114 (*P* < .05), or 118 (*P* < .01) days, respectively, compared to controls (Fig. 5D).

These data provide optimized CSI schedules for a range of preclinical MB models that can be applied for future studies. Furthermore, they illustrate that responses to CSI between MB models are varied and suggest every model should be optimized prior to undertaking preclinical studies.

### Tolerability of CSI

Routine monitoring of animals during and after CSI identified incisor overgrowth in approximately 25% of CSI-treated mice, including malocclusion arising from growth of additional teeth. This was managed by regular monitoring and incisor trimming under isoflurane anesthesia when required, and softened chow (normal chow moistened with water) was provided to ensure adequate nutrient intake. Radiation therapy–induced dental effects have been reported previously in irradiated Wistar rats.^29^ Despite irradiation of the head with both WBIR and CSI, this tooth phenotype was observed only in CSI-treated mice, reflecting the longer posttreatment survival of these animals (mice in WBIR cohorts reached humane endpoints due to spinal metastasis before late dental toxicities developed). Given CSI involves irradiation of the thoracic, lumbar, and cervical spine, we sought to determine if this protocol caused hematological changes. Weekly hematological monitoring over 6 weeks revealed no significant changes in red blood cells, hemoglobin, platelets, or neutrophils, and no clinical signs of anemia or thrombocytopenia (Figure E4). Mucositis was not observed in any treatment group despite the soft palate receiving incidental dose during head irradiation.

### DNA damage response pathway activation and extent of cell cycle arrest predict response to radiation therapy

To better understand these different responses to CSI across MB models, we studied the biological effects of radiation in orthotopic D425 and D283 G3 MB xenografts. These models were selected because they are widely used, exhibit similar median survival times in mice (16 days D425, 24 days D283), but had opposing responses to CSI (Figs 4A and 5A).

**Figure 6:**
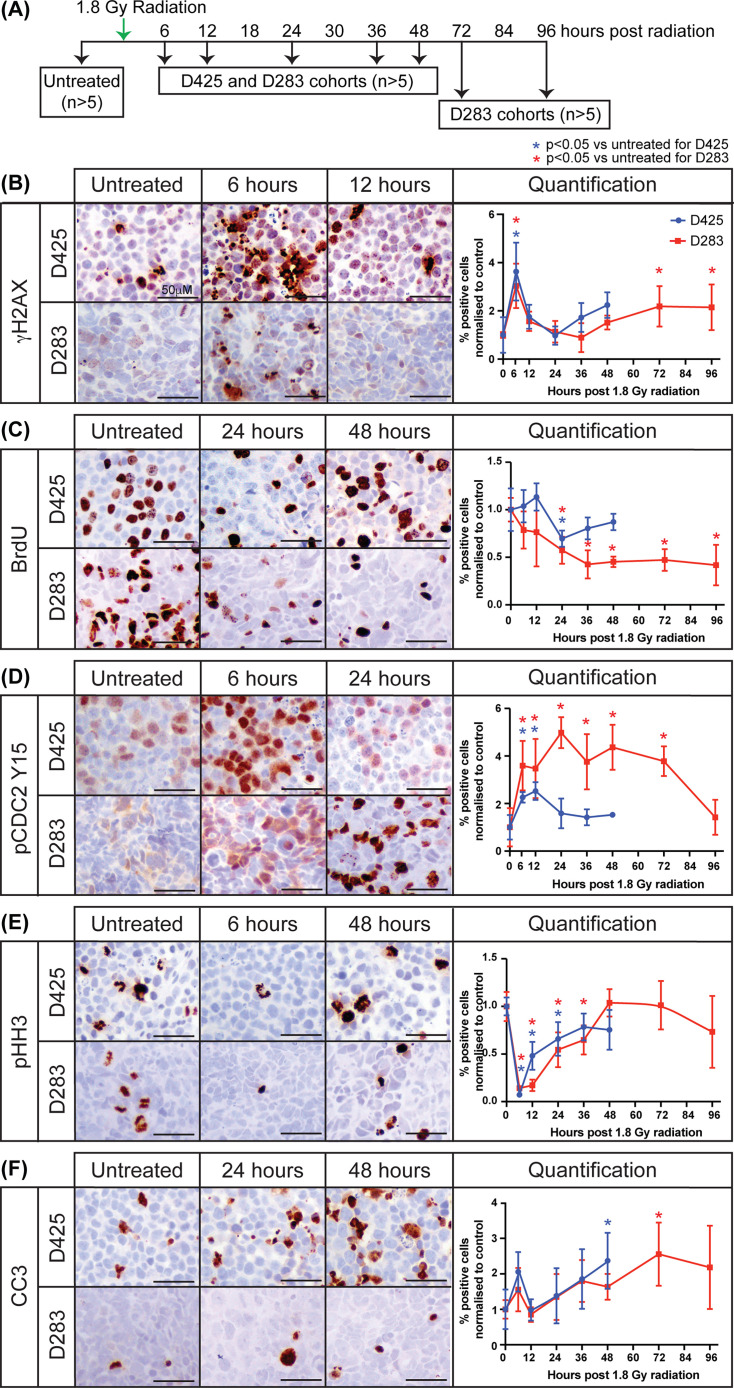
The extent of radiation-induced cell cycle arrest in MB xenografts inversely correlates with response. (A) Experimental schema for mice with orthotopic D425 or D283 MB xenografts. (B-G) Representative images (positive cells in *brown* with hematoxylin counterstain (*blue*), scale bar = 50 μm) and quantification (D425, *blue*; D283, *red*) of IHC is shown for (B) γH2AX, (C) BrdU, (D) pCDC2, (E) pHH3, and (F) CC3. Graphs indicate mean ± standard deviation. Significant differences (*, *P* < .05) compared to untreated controls were determined using 1-way ANOVA, with Dunnett’s multiple comparison test. *Abbreviations:* ANOVA = analysis of variance; IHC = immunohistochemistry; MB = medulloblastoma.

Tumor-bearing mice were treated with a single 1.8 Gy dose of CSI, and tissue was harvested at multiple time points for immunohistochemical assessments (Fig. 6A). It is well established that radiation induces DNA damage.^30^ Consistent with this, a sharp increase in γH2AX abundance was observed in D425 and D283 tumors within 6 hours of CSI which returned to baseline levels within 12 to 24 hours (Fig. 6B).

Upon DNA damage, cells typically trigger the DNA damage response pathway, leading to cell cycle arrest to enable DNA repair or apoptosis if irreprarable.^31^ To examine cell cycle activity, mice were injected with BrdU just prior to euthanasia to mark cells undergoing active DNA synthesis. In D425 tumors, BrdU incorporation was reduced 24 hours postradiation but appeared restored within 36 hours, indicating transient S-phase arrest. In D283 tumors, BrdU incorporation was also reduced 24 hours postradiation, however this persisted for up to 96 hours indicating prolonged S-phase arrest in this model (Fig. 6C). Radiation also induces G2 arrest^31^ and, when active, the DNA damage response should act to phosphorylate CDC2 and inhibit the CDC2/Cyclin B complex to block mitosis. Phosphorylated CDC2 was increased by 6 hours in both D425 and D283 xenografts confirming CSI induced cell cycle arrest. Of note, whereas this returned to control levels within 24 hours in D425 tumors, elevated levels in D283 tumors persisted up to 72 hours posttreatment, returning to baseline by 96 hours (Fig. 6D). Consistent with G2 arrest, there was a sharp reduction in mitosis, marked by phosphorylated histone H3 (pHH3) in both models after radiation, which slowly returned to baseline by 36 hours in D425 xenografts, but was delayed to 48 hours in D283 tumors (Fig. 6E). Apoptosis was not extensively observed after a single fraction of radiation, but cleaved caspase 3 (CC3) was transiently increased at 48 hours in D425 tumors, which was not observed until 72 hours in D283 tumors (Fig. 6F). Overall, a single dose of radiation appeared to only briefly stall cell cycle progression in D425 xenografts, but had a prolonged effect on D283 cells in vivo, which correlates with our observed effects of CSI on survival.

## Discussion

Radiation therapy is an essential component of clinical management for MB and other pediatric brain cancer; however, it causes long-term toxicities.^5^^,^^6^ There remains a compelling need to incorporate radiosensitizing agents clinically that enable dose reduction without compromising therapeutic efficacy. This will be best achieved by preclinical testing for such agents, to identify optimal radiosensitizers for clinical trial recommendation. One major criticism of preclinical research is the fact that novel agents that perform well preclinically have often failed to translate into favorable clinical outcomes. A possible reason is that many preclinical studies investigating radiosensitizers do not accurately mimic the dose or schedule of clinical radiation therapy protocols. Most studies to date use methods that lack accurate tissue targeting, delivering whole body or irradiation of the head with the body shielded using lead and often deliver a single dose (2-20 Gy)^32^, ^33^, ^34^, ^35^; it is conceivable that failure to mimic clinical schedules, and in turn not replicating the subsequent cellular biological responses, may lead to inaccurate preclinical outcomes. Thus, we sought to establish image guided radiation therapy protocols for orthotopic MB models using an X-RAD SmART platform which mimics clinical fractionation protocols, and can better recapitulate biological responses.

We took a cautious approach to establishing fractionated radiation therapy methods, beginning with focal and WBIR. Although not sufficient for tumor control in MB models, focal protocols may facilitate the incorporation of a posterior fossa boost to even more accurately mimic clinical protocols, or can readily be applied in studies involving less-infiltrative pediatric brain tumors where fractionated focal radiation therapy is clinically appropriate. Here, beam placement for focal irradiation was limited to the implantation coordinates as CBCT imaging cannot distinguish tumor from normal brain; however, software enabling coregistration of MRI and CBCT images would increase accuracy for such studies in future. WBIR did extend survival in MB models, however mice required euthanasia due to spinal metastasis-related morbidity. Given the preclinical evaluation of potential radiosensitizing drugs can only be achieved if all tumor cells are irradiated, we concluded CSI is the optimal method for such studies.^36^

Other preclinical fractionated CSI methods have been described prior to this study, although this is the first developed using the X-RAD SmART system. Huang et al^37^ used a high-throughput lead shielding approach, delivering CSI and a focal boost; however, targeting accuracy and impact of off-target doses to surrounding organs was not described. Morrissy et al^15^ used a 20 × 20-mm square collimator to target the brain via 2 laterally opposed beams, together with a 5 × 50-mm custom-built collimator to target the spine, with the mouse placed supine on the stage and irradiated from below, although spinal irradiation was not performed on a 5-on-2-off schedule. The requirement of a custom-built or multileaf collimator limits implementation of this protocol in other institutions. A third example of preclinical CSI delivered using a SARRP has also described.^16^ In this, mice were positioned prone on the stage with the spine straightened. The brain was treated with a single beam arc from −90 to 90° and the spine was irradiated using posterior-to-anterior beam with 2 separate isocenters in the spinal cord, similar to Morrissy et al^15^ No toxicity was reported and Monte Carlo dose calculations indicated low levels of off-target radiation to the thorax and abdominal organs using this device. Of note, this methodology cannot be replicated on the X-RAD-SmART 225Cx system because of the inability of the gantry to rotate through 0°. Instead, we attempted a similar method to deliver radiation therapy to the spine with a rotating x-ray beam and a 5-mm circular collimator; however, mice exhibited unacceptable weight loss suggesting this technique is not ideal for studies where long-term survival of mice is expected.

Clinically, MB patients receive 23.4 or 36- to 39.6-Gy fractionated CSI, with a boost to 54 Gy at the tumor bed.^5^^,^^6^ CSI alone is rarely curative clinically, and disease often relapses in a different part of the central nervous system.^38^ Conversely, most CSI-treated tumors recurred locally in the models tested here. This was similar to Smith et al*,*^16^ likely because of our models not undergoing surgical removal of the tumor bulk as occurs in patients, and/or the lack of focal boost to the tumor bed. In terms of cumulative dose in this and previous studies, the amount of CSI capable of curing mice with MB, even in the absence of surgery, was much lower compared to patient doses.^15^^,^^16^^,^^37^ Moreover, in order to develop preclinical radiation therapy protocols to assess radiosensitizing strategies for MB, we aimed to define a dose that would extend survival but not cure mice. Eighteen to 20-Gy CSI (delivered over 9-10 fractions) was optimal for D425 and SU-MB002 xenografts, but was curative for D283 MBs. Smith et al^16^ also described a patient-derived model (HD-MB03) where 20-Gy CSI was curative. Although these models are all derived from G3 MB and have similar median survival (16-28 days), it appeared that the radiosensitive models were wildtype (WT) for *TP53*, whereas the more radioresistant models have *TP53* disruption.^17^, ^18^, ^19^, ^20^, ^21^ Consistent with this, our investigation of in vivo cell cycle status using IHC revealed striking differences between 2 cell lines, with prolonged S-phase arrest and slow return to cell cycling in CSI sensitive *TP53* WT D283 cells, compared to *TP53* mutated, less CSI sensitive D425 cells. These observed effects are consistent with the known functions of p53, where WT p53 normally mediates cell cycle arrest to allow for DNA repair,^39^ whereas mutant forms of p53 can increase tolerance to DNA damage, increase DNA repair mechanisms and reduce the apoptotic response leading to radiation resistance.^15^^,^^40^, ^41^, ^42^

In addition, we observed that the dose of CSI required to increase survival but not cure PDOX models of MB was significantly less than clinical radiation therapy doses and was also less than the dosages required to control tumors derived from long-term cultured MB cell lines or Myc/p53^DD^ murine MB cells.^15^^,^^16^^,^^37^ Consistent with the way *TP53* status correlated with radiation-induced cell cycle impacts in D425 and D283 xenografts, we note that the CSI sensitive G3 and G4 MB PDOXs used here (TK-MB-913 and MED211FH) have *TP53* WT, whereas the MED813FH SHH PDOX, which was less sensitive to CSI, is *TP53* mutated.^21^^,^^43^ These studies confirm the critical role of p53 in mediating radiobiological responses in MB and suggests that knowing the *TP53* status may be useful to help researchers develop optimal CSI treatment schedules for other MB PDOX models in the future. Of note, Smith et al^16^ described that G3 PDOX models with WT *TP53* and SHH models harboring *TP53* mutation had similar responses to CSI, suggesting the possibility that PDOX models in general are more radiosensitive than cell lines and this may be irrespective of *TP53* status.^41^ We note that our γH2AX IHC was used as a high-throughput readout of DNA damage response activation across many tumors and time points, and that the chromogenic IHC platform used here does not reliably distinguish between focal and pan-nuclear γH2AX patterns. Higher-resolution immunofluorescence imaging would be required to discriminate these patterns and assign them to distinct underlying biology (focal foci reflecting double-strand breaks; pan-nuclear staining associated with replication stress or preapoptotic cells) and represents a valuable extension of the present methods-focused study.

The overall lower CSI dosage required to mimic clinical outcomes, in terms of both dose per fraction and number of fractions, is a limitation of the murine models. This may be a factor of time, since patient relapses following CSI are measured in years, whereas the short lifespan of the mouse, as well as practical, ethical, and experimental limitations, restricts the time that animals can be followed in preclinical experiments. Growth kinetics, in part determined by cell cycle length, may also play a role, especially in PDOX models which are typically derived from treatment-naïve samples and are generally slower growing compared to long-term MB cell lines, that have likely selected inherently faster growing clones over many years in culture.^44^ Another contributing factor to diversity in response to radiation across different models may be that there is less genetic heterogeneity in PDOX and cell line models, compared to genetically engineered mouse models and primary MB.^45^ To better dissect these mechanisms, future studies could preclinically investigate radiobiological responses, and DNA repair mechanisms, coupled with genetic methods to study clonogenicity following single and multifraction CSI doses in MB models. Such studies would be critical to understand if radio-resistance in MB is subclonal, and to define molecular mechanisms contributing to treatment resistance and disease relapse.

This CSI protocol represents the first to be described using the X-RAD-SmART 225Cx system without custom collimation and we have defined optimal CSI schedules for multiple preclinical models of MB. This method requires approximately 10 minutes per mouse, making large preclinical studies feasible. Moreover, the schedules ranging from 1 to 4 weeks provide a sufficient treatment window to overlay novel therapies and assess both efficacy and side effects that may not be evident using single dose methods of radiation therapy. The efficacy of the poly (ADP-ribose) polymerase (PARP) inhibitor veliparib was recently evaluated using our strategy, demonstrating that combining CSI with veliparib only minimally increased survival and does not warrant clinical translation.^28^ High-fidelity preclinical studies like these are pivotal for accelerating progress in the field. By enabling rapid and rigorous evaluation of multiple therapeutic candidates, they streamline the translation of only the most promising radiosensitizing approaches, ultimately driving meaningful improvements in patient outcomes.

## Conclusions

Here, we established image guided, multifraction irradiation protocols for preclinical MB models using the X-RAD-SmART 225Cx system and identified CSI as the most clinically relevant approach to replicate treatment patterns and regrowth. Sensitivity to CSI varied markedly across models: MB PDOX and *TP53* wildtype G3 cell lines were more responsive than *TP53*-mutated G3 cell lines and murine-derived models. Importantly, CSI doses required to approximate clinical outcomes were consistently lower than those used in patients, underscoring the need for careful dose translation. Although immunohistochemical markers of DNA damage response and cell cycle dynamics may hold predictive value, variability across MB models highlights the complexity of treatment response. These findings reinforce the necessity of rigorous dose optimization in each new preclinical model before embarking on large-scale studies, ensuring translational relevance and improving the predictive power of preclinical research.

## Disclosures

The authors declare that they have no known competing financial interests or personal relationships that could have appeared to influence the work reported in this paper.

## Appendix Supplementary materials

Supplementary Figures_resub_clean.docx Image, application 1

Supplementary Material resub clean.docx Image, application 2

Supplementary Table S1.docx Image, application 3

## Footnotes

Supplementary material associated with this article can be found in the online version at doi:10.1016/j.adro.2026.102114.
