# Supplementary material for: Establishing a Clinically Relevant Radiation Therapy Method for Preclinical Medulloblastoma Research
Source: Adv Radiat Oncol. 2026 Jun 10;11(11):102114. doi: 10.1016/j.adro.2026.102114 (PMC13400247; doi:10.1016/j.adro.2026.102114)
Supplement: Supplementary Figures_resub_clean.docx [file mmc1.docx]

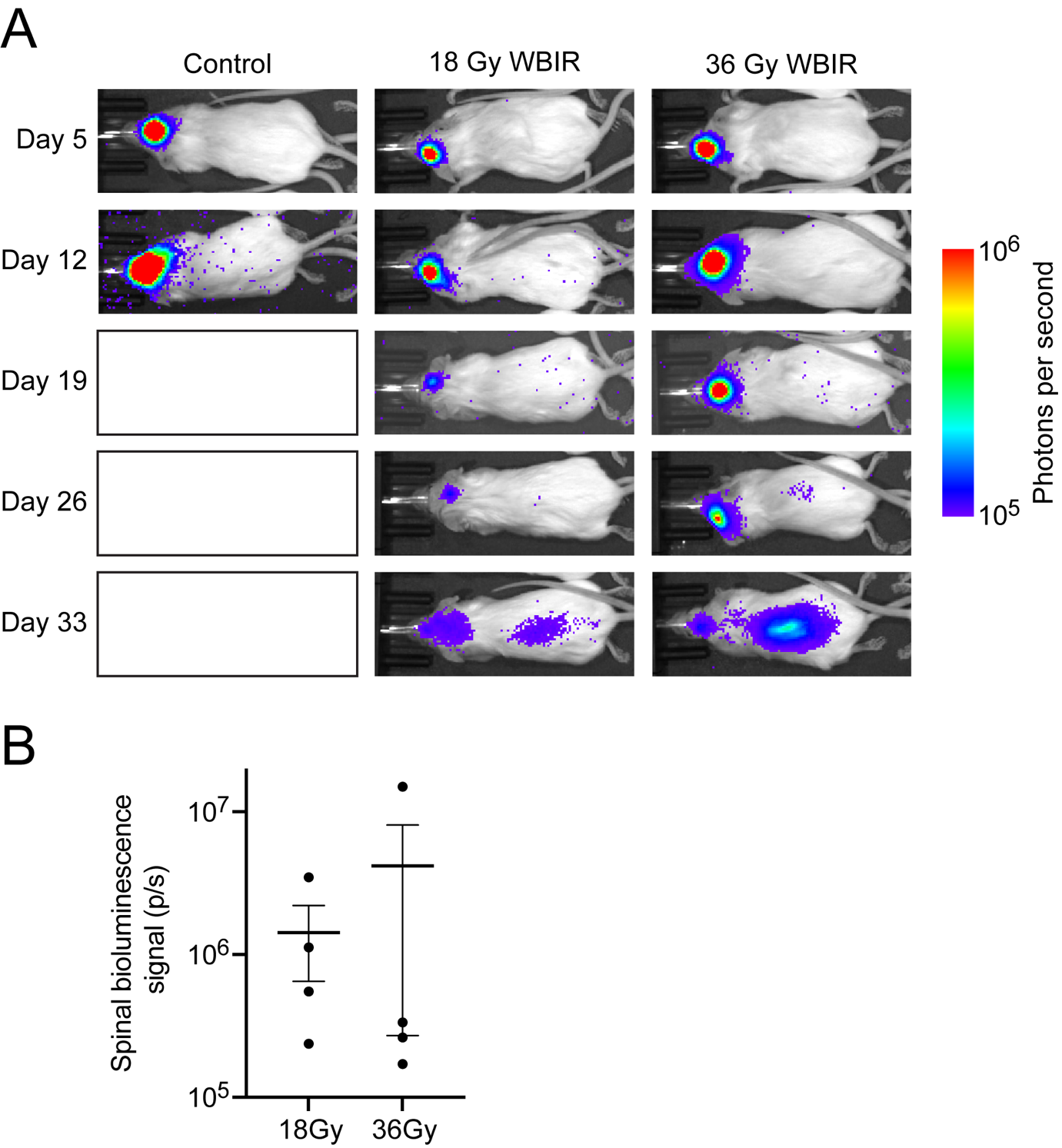


Supplementary Figure S1 – Whole brain irradiation reduces brain tumour burden but allows escape of metastatic cells to the spinal cord. (A) Representative bioluminescence images of mice in the control, 18 Gy WBIR, and 36 Gy WBIR groups. The Day 5 image was acquired before the start of treatment later that day. Spinal metastases are visible from Day 26 and are substantial at Day 33. Color scale indicates radiance. (B) Maximum luciferase radiance over the spine was measured (excluding head region) and plotted for each treatment group at the Day 33 timepoint. Error bars show mean ± standard deviation.


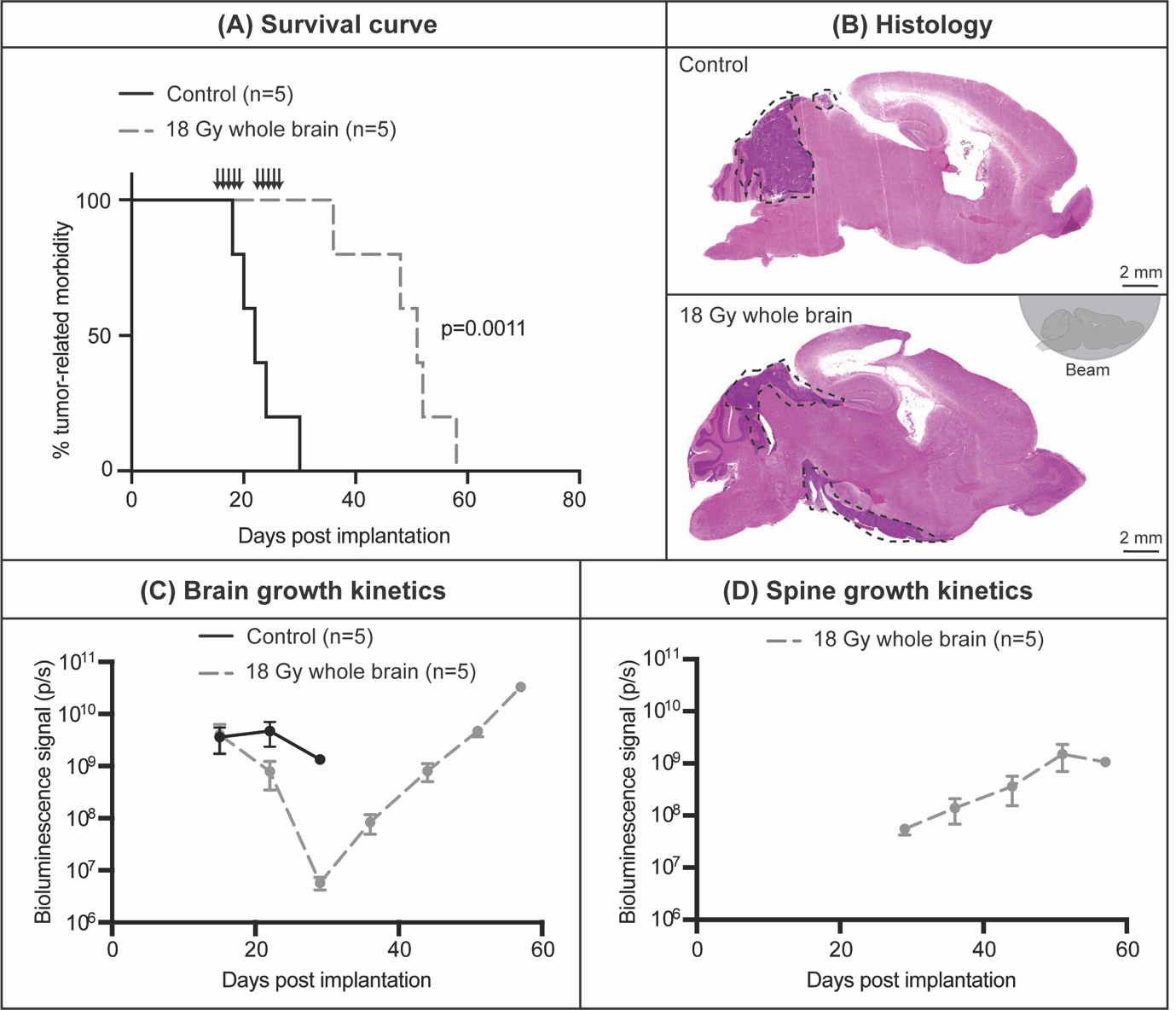


Supplementary Figure S2 - Whole brain irradiation increases survival of mice with SU-MB002 MB. (A) Kaplan-Meier survival curves for mice implanted with SU-MB002 MB cells into the cerebellum that were treated as controls or that received 18 Gy whole brain irradiation (fractions are marked by the downward arrows). Number of mice is indicated (*n*). (B) Representative H&E-stained sections of SU-MB002 tumors from untreated control mice, and mice following 18 Gy fractionated whole brain irradiation, at humane endpoint. Tumors are indicated (*dashed line*) and the inset illustrates the treatment field (*dark grey*). (C) Bioluminescence signals (photons/ second; p/s) from the brains of the above mice over time. (D) Bioluminescence signals (photons/ second; p/s) from the spine of the above mice over time.


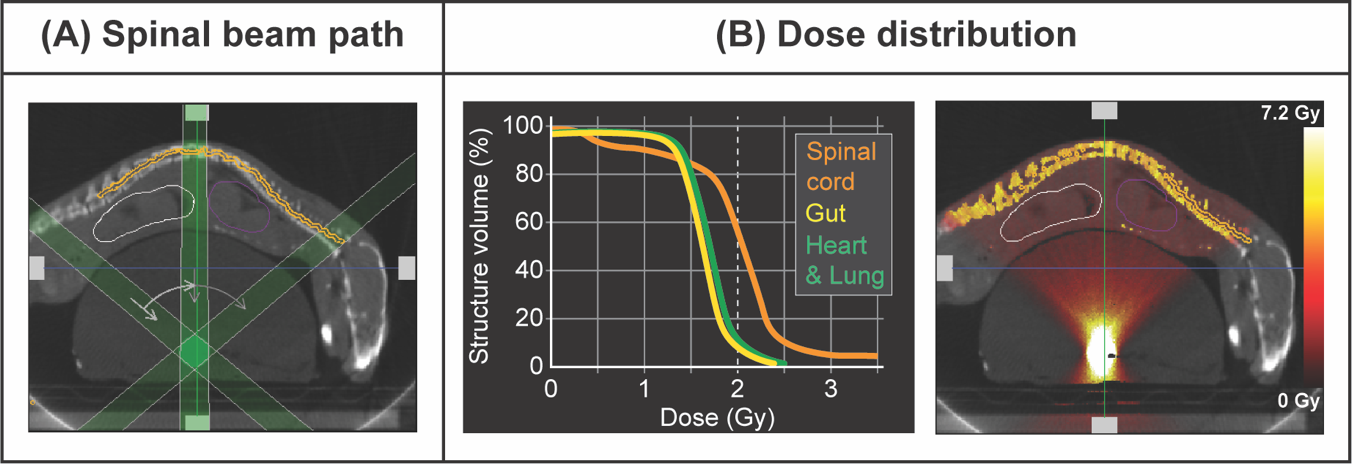


Supplementary Figure S3: Alternative CSI protocol causes unacceptable weight loss. This treatment plan utilized the whole brain irradiation method described in Figure 1C, coupled with spinal irradiation. (A) CBCT image of an anesthetized mouse positioned over a curved support illustrating the starting and finishing positions of a rotating x-ray beam delivered via a 5 mm diameter circular collimator (*green*). The direction of the rotations are indicated by white arrows. Areas defined manually for dose calculations are marked as follows: spinal cord (*orange*), gut (*white*), and heart/lung (*purple*). (B) Dose-volume histogram shows approximately 85% of the spinal cord received 1.8 Gy, and approximately 60% received 2 Gy (*dashed line*). Approximately 90% of the gut and heart/lung received 1.5 Gy. The calculated dose is illustrated at the right as a yellow-red heat map overlaid on the CBCT image.


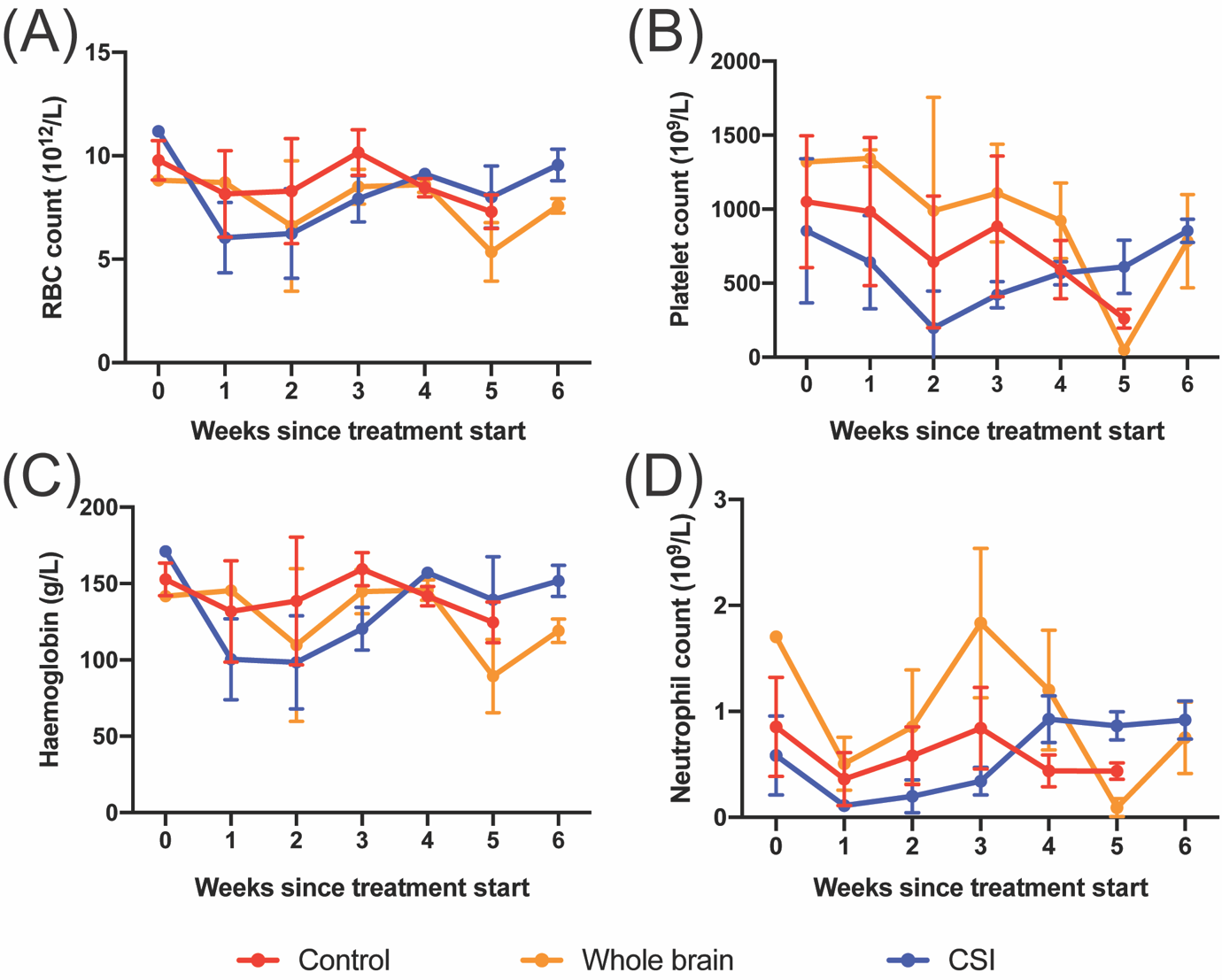


Supplementary Figure S4: Hematological parameters from tumor-bearing NRG control mice (*red*), or following treatment with whole brain irradiation (*orange*) or CSI (*blue*). Blood was assessed for (A) Red blood cells (RBC), (B) platelets, (C) hemoglobin, and (D) neutrophils. No significant differences were observed between treatment groups at any timepoint. Data is shown as mean ± standard deviation from 3 mice per timepoint.
