# Supplementary material for: Establishing a Clinically Relevant Radiation Therapy Method for Preclinical Medulloblastoma Research
Source: Adv Radiat Oncol. 2026 Jun 10;11(11):102114. doi: 10.1016/j.adro.2026.102114 (PMC13400247; doi:10.1016/j.adro.2026.102114)
Supplement: Supplementary Material resub clean.docx [file mmc2.docx]

## Supplementary Material

## Supplementary Methods

### Mouse strains

NOD/*Rag1*^-/-^ and C57BL/6J/ *Rag1*^-/-^ mice were acquired from The Jackson Laboratory and maintained at the Bioresources Centre of The Kids Research Institute Australia. NOD/*Rag1*^-/-^/*Il2g*^-/-^ (NRG) and BALB/c nude mice were acquired from Ozgene Animal Resource Centre (Australia) and were allowed to acclimatize for at least 72 hours prior to use. All mice had access *ad libitum* to food and water, with a 12-hour light/dark cycle. Sample sizes of at least 4 per group were determined to be sufficient to detect a 30% increase in survival between groups with a probability (power) of 0.8 and a type I error rate of 0.05.

### Cell lines and PDOX models

D425 G3 human MB cells were cultured in modified IMEM (#A10489-01, Gibco) supplemented with 1% GlutaMAX (#35050-061, Gibco), 10% fetal bovine serum (FBS, Cell Sera), and 10 µM HEPES (#15630-080, Gibco).[^1^](#_ENREF_1) D283 G3 human MB cells were cultured in DMEM (#11960069) supplemented with 1% GlutaMAX and 10% FBS.[^2^](#_ENREF_2) SU-MB002 G3 human MB cells were maintained *in vivo* via orthotopic xenograft in mice, then cultured briefly as previously described[^3^](#_ENREF_3) prior to implantation. These cell lines were incubated at 37°C in 5% CO_2,_ confirmed mycoplasma-free using a MycoAlert™ Mycoplasma Detection Kit (Lonza) and verified using STR profiling (2019, Genetic Resources Core Facility, Johns Hopkins University).

TK-MB913 G4, MED211FH G3 and MED813FH SHH PDOX models cannot be cultured *in vitro* and were maintained *in vivo* via serial orthotopic xenografts in NOD/*Rag1*^-/-^ mice.[^4^](#_ENREF_4) Myc/p53^DD^ mouse MB cells[^5^](#_ENREF_5) were maintained via serial orthotopic allografts in C57Bl/6 or NOD/*Rag1*^-/-^ mice.

D425, SU-MB002 and TK-MB913 cells were retrovirally transduced using pCL20-MSCV-GFP-ires-Luc2. D283 cells were retrovirally transduced with MSCV-ires-pacLuc2. Viral constructs and packaging plasmids were kindly provided by Arthur Nienhuis and Richard Williams of St Jude Children’s Research Hospital, USA.

Summaries of the details for each model are described in **Supplementary Table S1**.

### Radiotherapy specifications

The X-RAD SmART (Precision X-ray, North Branford CT) can deliver radiotherapy via a focal-spot X-ray tube mounted on a rotating gantry. X-ray energies up to 225 kVp can be generated and delivered to an isocenter 30.6 cm from the source. The Monte Carlo dose calculation performed within SmartPlan requires a CBCT scan of the target animal and pre-established phase space files for each collimator used. In designing each treatment plan, we aimed to achieve accurate calculation of dose throughout the whole animal anatomy, uniformity of dose to the brain and spinal cord, and reduce normal tissue exposure to limit animal impost. In addition, to facilitate application of the technique in preclinical studies that involve large animal numbers, we endeavored to develop a protocol that would be easy to setup, has minimal anesthesia time, the treatment field could be positioned using rapid imaging, and that minimized the need for multiple collimators. Since any changes to animal positioning during treatment would require re-imaging and repeated dose calculations, we also aimed to avoid moving the animal between treatment beams. Lastly, limitations of the system also needed to be taken into account. Specifically, the source in the XRAD SMART is unable to rotate continuously through a gantry angle of 0°, corresponding to when the source is directly above the target. As such, treatments that required the source to rotate above the animal were more time consuming to administer.

Mice were anaesthetized outside the device in an induction chamber with 4-5% isoflurane using a SomnoSuite® System, and were maintained via delivery of 1-2% isoflurane using a nose cone within the XRAD SMART. Animals were placed in a feet-first prone position on a carbon-fibre stage, and immobilized using non-stick sports tape. Treatment planning was achieved using 3-dimentional CBCT images of each target animal, acquired using PilotCal software (v1.8, Precision X-ray) and a 60 kV high current beam, 2 mm Al filter with small spot size producing images with 0.1 mm resolution[^6^](#_ENREF_6) Images are imported into SmartPlan for dose calculations was undertaken using the EGSnrc Monte Carlo code based on medium information derived from CBCT images and pre-established phase space files for each collimator[^7^](#_ENREF_7). Determination of the device output for absorbed dose calculations has been previously established according to the protocol of the American Association of Physicists in Medicine[^6^](#_ENREF_6).

The stage, nose cone and sports tape were all included in the CBCT image acquisition and incorporated in determining the dose distribution calculations which involved: i) manual definition of tissue types within the CBCT images, ii) delineating target volumes and organs-at-risk, iii) positioning beam isocenters and beams, iv) prescribing dose to beam isocenter locations, v) calculating dose with a set calculation of uncertainty at isocenter of 2%. In this study, a series of collimators were used to shape the beam (10x10mm square, 40x40mm square or 25 mm diameter circle). The software is limited to calculating the dose at the beam isocenter, which is ideally located in tissue. However, due to the size of experimental mice and the dimensions of the collimators, in some cases the beam isocenter was positioned in the air above the animal. In these cases, a “faux” target was drawn and defined as tissue in the CBCT images to facilitate accurate dose calculation. To ensure each treatment plan was suitable, the calculated dose distribution was assessed by checking that the dose-volume distribution within the actual target tissue was at the desired dose. Exposure of organs-at-risk were also documented.

Treatment delivery was controlled using PilotCal software, using a 225 kV beam, 0.32 mm Cu filtration with a large source size at 13 mA tube current, delivering approximate dose-rates of between 2.8-3.5 Gy/min at the isocenter. The half value layer of our device was determined to be 1.05 mm Cu[^8^](#_ENREF_8). The spatial and dosimetric accuracy of the system has previously been established[^8-10^](#_ENREF_8), and the device is regularly checked via an institutional quality assurance program. Doses are reported as dose-to-medium. Each of the four dose plans were designed so that at least 90% of the target tissue (tumor) received 1.8 Gy per fraction, as demonstrated in Figures 1-3.

Slight modifications were required for each different treatment field. For focal irradiation, a CBCT was acquired and a new dose plan was defined prior to every treatment for each mouse. This was performed to ensure the treated area was accurately positioned relative to the implantation site (identified via CBCT by the hole in the skull that remained following implantation). Approximate treatment time per mouse (including positioning animal on the stage, imaging and treatment planning) was 20 minutes. For the other treatment plans (square beam, whole brain, or CSI), a single treatment plan on the first animal was established, and then applied across all animals in that cohort. To ensure correct placement of the beam, fluoroscopic imaging was performed with the collimator in place so that skeletal landmarks could be used to position the field by moving the animal holding stage manually. This also served to safeguard against accidental overexposure at the junctions when multiple fields were used in the CSI protocol. Approximate treatment time per mouse was 4 minutes for square beam and whole brain irradiation, and 9 minutes for CSI.

## Supplementary Results

### An alternative CSI protocol resulted in severe toxicity precluding further use

In development of different methods for the delivery of CSI to mice, we tested a method that took advantage of the rotating gantry of the XRAD SmART. To irradiate mice using this method, a CBCT scan was acquired, and anatomical structures were defined. 1.8 Gy radiotherapy was first delivered to the brain using a 25 mm diameter circular collimator according to the whole brain treatment plan described in the main methods. Mice were allowed to recover, and in a separate anesthesia session, the spine was irradiated from above the animal using a 5 mm diameter circular collimator, rotating along the spine in a rostral-to-caudal direction. To facilitate even dose distribution to the spine, animals were positioned over a 4.5 cm diameter curved support by placing the front legs underneath the chest with the hind legs straightened. The mouse was gently secured on the support with non-stick sports tape. The stage and animal were positioned to ensure the spine was directly in the beam path and the length of the spine aligned with the curve of the rotating gantry in the XRAD. As mentioned above, a limitation of the XRAD SmART 225C is that the gantry and x-ray source cannot move through the 0° position. As such, to irradiate the entire spine, two separate arc beams were required to deliver therapy to each side of the curved support. The first x-ray beam started at 0° and continued delivering radiation while the gantry rotated to approximately 40° where the cervical spine connects with the skull. The gantry was then rotated under the animal, and the second treatment beam delivered radiotherapy starting from approximately 320°, just before the iliac crest of the pelvis, until the gantry reached 360°. The isocenter of these two spinal beams is located within the curved support (**Supplementary Figure S3A**).

To calculate the speed of the rotating x-ray beam such that the spine would receive 1.8 Gy, the spinal cord was contoured carefully using SmART-Plan software to exclude all bone. It was determined that delivery of 5 Gy to the isocenter resulted in the delivery of 1.8-2 Gy to 90% of the spinal cord (**Supplementary Figure S3B**, orange line). Assessment of the radiation exposure to tissues outside the spinal cord, along the midline of the animal indicated that there was significant exposure to the abdominal organs along the midline (**Supplementary Figure S3B,** yellow and green lines).

To determine if this protocol was suitable for mice, it was assessed using non-tumor bearing NRG mice. 1.8 Gy whole brain irradiation fractions were delivered to the brain in two 7-day cycles consisting of 5 days on, 2 days off. The spine was irradiated twice per week (on days 1 and 4 of each 7-day cycle), mimicking the approach described previously by Morrissy *et. al*[^11^](#_ENREF_11). Unfortunately, unacceptable toxicity was observed in all mice treated. The onset of side effects were observed at the end of cycle 1, after mice had received five 1.8 Gy fractions to the brain, and two 5 Gy fractions to the spinal cord. Symptoms included weight loss and ruffled fur. Treatment was stopped and supportive care immediately provided, including soft food, nutritional supplement gel and enrofloxacin in the drinking water. Despite this, mice continued to lose weight, and the cohort was euthanized at a predefined ethical endpoint (20% weight loss). Necropsy showed the absence of food in the stomach and little digesta in the intestines. All other organs appeared macroscopically normal. Given these adverse effects, this rotating CSI treatment plan is not recommended for preclinical use. For the CSI method described in **Figure 1D**, details about the non-dose limiting toxicities observed (incisor overgrowth and absence of hematological changes) are provided in the main text. Hematology data are shown in **Supplementary Figure S4**.

## Supplementary References

1. Bigner SH, Friedman HS, Vogelstein B, Oakes WJ, Bigner DD. Amplification of the c-myc gene in human medulloblastoma cell lines and xenografts. *Cancer Res*. Apr 15 1990;50(8):2347-50.

2. Friedman HS, Burger PC, Bigner SH, et al. Establishment and Characterization of the Human Medulloblastoma Cell Line and Transplantable Xenograft D283 Med. *Journal of Neuropathology & Experimental Neurology*. 1985;44(6):592-605. doi:10.1097/00005072-198511000-00005

3. Bandopadhayay P, Bergthold G, Nguyen B, et al. BET bromodomain inhibition of MYC-amplified medulloblastoma. *Clin Cancer Res*. Feb 15 2014;20(4):912-25. doi:10.1158/1078-0432.Ccr-13-2281

4. Brabetz S, Leary SES, Gröbner SN, et al. A biobank of patient-derived pediatric brain tumor models. *Nature Medicine*. 2018/11/01 2018;24(11):1752-1761. doi:10.1038/s41591-018-0207-3

5. Pei Y, Moore CE, Wang J, et al. An animal model of MYC-driven medulloblastoma. *Cancer Cell*. Feb 14 2012;21(2):155-67. doi:10.1016/j.ccr.2011.12.021

6. Ma CM, Coffey CW, DeWerd LA, et al. AAPM protocol for 40–300 kV x-ray beam dosimetry in radiotherapy and radiobiology. *Medical Physics*. 2001/06/01 2001;28(6):868-893. doi:10.1118/1.1374247

7. Kawrakow I. Accurate condensed history Monte Carlo simulation of electron transport. I. EGSnrc, the new EGS4 version. *Medical Physics*. 2000/03/01 2000;27(3):485-498. doi:10.1118/1.598917

8. Feddersen TV, Rowshanfarzad P, Abel TN, Ebert MA. Commissioning and performance characteristics of a pre-clinical image-guided radiotherapy system. *Australasian Physical & Engineering Sciences in Medicine*. 2019/06/01 2019;42(2):541-551. doi:10.1007/s13246-019-00755-4

9. Lindsay PE, Granton PV, Gasparini A, et al. Multi-institutional dosimetric and geometric commissioning of image-guided small animal irradiators. *Medical Physics*. 2014/03/01 2014;41(3):031714. doi:10.1118/1.4866215

10. van Hoof SJ, Granton PV, Verhaegen F. Development and validation of a treatment planning system for small animal radiotherapy: SmART-Plan. *Radiotherapy and Oncology*. 2013/12/01/ 2013;109(3):361-366. doi:<https://doi.org/10.1016/j.radonc.2013.10.003>

11. Morrissy AS, Garzia L, Shih DJH, et al. Divergent clonal selection dominates medulloblastoma at recurrence. *Nature*. 2016/01/01 2016;529(7586):351-357. doi:10.1038/nature16478
