# Supplementary material for: Establishing a Clinically Relevant Radiation Therapy Method for Preclinical Medulloblastoma Research
Source: Adv Radiat Oncol. 2026 Jun 10;11(11):102114. doi: 10.1016/j.adro.2026.102114 (PMC13400247; doi:10.1016/j.adro.2026.102114)
Supplement: Supplementary Table S1.docx [file mmc3.docx]

## Supplementary Table S1 – Summary of preclinical models and experimental conditions. Implantation site, cell number, mouse strain and sex, tumour subtype, *TP53* status and treatment start day for each medulloblastoma model used in this study.

| **Model** | **Tumor subtype** | ***TP53* status** | **Cell source** | **Cell number implanted** | **Treatment start day** | **Implantation site** | **Mouse strain** | **Mouse sex** |
| --- | --- | --- | --- | --- | --- | --- | --- | --- |
| D425 | G3 MB (cell line) | Mutant | Long-term *in vitro* cell culture | 5 × 10^5^ | 5 | Cortex | *BALB/c nude*  (Figure 2A) | Female |
|  |  |  |  |  |  |  | *NOD/Rag1*^−/−^  (Figures 2B, 3, S1) | Mixed |
|  |  |  |  |  |  |  | *NRG* (Figures 4A, 6) | Female |
| D283 | G3 MB (cell line) | Wild-type | Long-term *in vitro* cell culture | 5 × 10^5^ | 7 | Cortex  (Figure 6) | *NRG* | Female |
|  |  |  |  | 1 × 10^5^ |  | Cerebellum (Figure 5A) | *NRG* | Female |
| SU-MB002 | G3 MB (cell line) | Mutant | *In vivo* serial passage; brief culture prior to implantation | 5 × 10^5^ | 7 | Cortex  (Figure 4B, S4) | *NRG* | Female |
|  |  |  |  | 1 × 10^5^ |  | Cerebellum (Figure S2) | *NRG* | Female |
| Myc/p53^DD^ | G3 MB (murine allograft) | p53 dominant-negative | *In vivo* serial passage | 5 × 10^3^ | 5 | Cortex | *C57BL/6J/Rag1*^−/−^ (Figure 4C) | Mixed |
|  |  |  |  |  |  |  | *NOD/Rag1*^−/−^  (Figure 2C) | Mixed |
| TK-MB913 | G4 MB (PDOX) | Wild-type | *In vivo* serial passage | 1 × 10^5^ | 20, 30 | Cerebellum | *NOD/Rag1*^−/−^ | Mixed |
| MED211FH | G3 MB (PDOX) | Wild-type | *In vivo* serial passage | 1 × 10^5^ | 14, 21 | Cerebellum | *NOD/Rag1*^−/−^ | Mixed |
| MED813FH | SHH MB (PDOX) | Mutant | *In vivo* serial passage | 1 × 10^5^ | 38 | Cerebellum | *NOD/Rag1*^−/−^ | Female |

*MB, medulloblastoma; PDOX, patient-derived orthotopic xenograft; G3, Group 3; G4, Group 4; SHH, Sonic Hedgehog; NRG, NOD/ Rag1*^−/−^/*Il2rg*^−/−^*. TP53 status as previously published (citations as in main text). NOD/Rag1^−/−^ and C57BL/6J/Rag1^−/−^ mice were obtained from The Jackson Laboratory; NRG and BALB/c nude mice were obtained from Ozgene Animal Resource Centre.*
